# Heart rate and sub-blubber temperature in harbour seals (*Phoca vitulina*) measured during the moult and post-moult using implantable loggers

**DOI:** 10.1007/s00360-026-01689-6

**Published:** 2026-07-20

**Authors:** W. D. Paterson, L. L. Chaise, S. E. Moss, R. Milne, C. Gilbert, J. I. Currie, D. Thompson, D. J. McCafferty

**Affiliations:** 1 Sea Mammal Research Unit, Scottish Oceans Institute, University of St. Andrews, Fife, St. Andrews, KY16 8LB Scotland, UK; 2 UMR 7179 Laboratoire MECADEV CNRS/MNHN, 1 Avenue du Petit Château, 91800 Brunoy, France; 3 Ecole Nationale Vétérinaire d’Alfort, Ethologie, 7 Avenue du Général de Gaulle, 94700 Maisons-Alfort, France; 4 School of Computing, Engineering and the Built Environment, Edinburgh Napier University, Merchiston Campus, Colinton Road, Edinburgh, EH10 5DT Scotland, UK; 5 Scottish Centre for Ecology and the Natural Environment, School of Biodiversity, One Health and Veterinary Medicine, University of Glasgow, Rowardennan, Glasgow, G63 0AW Scotland, UK

**Keywords:** Moult physiology, Pinniped, Heart rate, Body temperature, Implanted logger

## Abstract

The moult represents an important seasonal change in the physiology of phocid seals. However, physiological measurements are challenging due to the impracticalities of instrumenting moulting animals. The aim of this study was (i) to assess the feasibility of a long-term deployment of fully implantable loggers to measure heart rate (HR) and sub-blubber temperature (T_b_) in harbour seals (*Phoca vitulina*) and (ii) to compare HR and T_b_ during moult and post-moult periods. Loggers were surgically implanted on an adult and sub-adult harbour seal in captivity. Animals healed quickly from surgery and loggers provided 65 days of continuous data before removal of the device. Results showed that in the adult when hauled out and in the water HR was lower during the moult compared with the post-moult. For the sub-adult, HR while in the water was also higher during the post-moult, however, when hauled out there was no difference in HR between the moult and post-moult periods. When hauled out, T_b_ was greater for both animals during the moult compared with the post-moult. During haulout events, T_b_ increased over time in both periods and T_b_ in both animals was lower during post moult compared to the moult period. In the sub-adult, T_b_ decreased with time in the water during the moult but showed little change during the post-moult period, whereas no temporal change was evident in the adult during either period. This study demonstrated that fully implantable loggers are a useful research tool allowing measurement of physiological parameters that are otherwise difficult to measure in pinnipeds. Physiological measurements suggested that the requirement to maintain higher skin temperature for hair growth during the moult was offset by lower metabolism to save energy in water, and on land in the case of adults. However, a decrease in body temperature with time in water in the sub-adult indicated that a smaller body size and less well-developed blubber layer is not as effective in reducing heat loss compared to adults. Future physio-logging on pinnipeds throughout their annual lifecycle may therefore benefit from similar techniques used to implant loggers.

**Supplementary Information:**

The online version contains supplementary material available at 10.1007/s00360-026-01689-6.

## Introduction

Physiologging has increased our understanding of diving physiology in marine mammals by providing data on changes in heart rate as animals dive to depth (Thompson and Fedak 1993; Williams et al. 2015; Elmegaard et al. 2016) and how heat loss is minimised in the marine environment (Boyd 2000; Willis et al. 2005; Hindle et al. 2015; Favilla and Costa 2020). Phocid seals that periodically haulout on land to rest, breed and moult, must also adapt physiologically to the terrestrial phase of their annual life cycle. A thick insulating blubber layer allows seals to conserve heat while at sea (Molyneux and Bryden 1978; Khamas et al. 2012) while on land, seals may be exposed to ambient conditions in which a thick insulating layer is not always required. Thermoregulation is achieved by increasing blood flow to the skin surface, by-passing the blubber and creating thermal windows through which excess heat is lost (Erdsack et al. 2012; Codde et al. 2016; Favillla et al. 2022). Phocids are highly adapted to control perfusion of blood to different parts of the body while diving and when on land. Knowing how circulatory changes, reflected in heart rate, are related to changes in body temperature will improve our understanding of thermoregulation associated with the transition between the marine and terrestrial environment.

Two important phases in the annual lifecycle of phocid seals that are characterised by greater time spent on land are the breeding season (Hindell and Burton 1988; Cordes and Thompson 2015) and the moult (Thompson et al. 1989; Boyd et al. 1993). During the moult, the shedding and renewal of hair requires elevation of skin temperature which can usually only be achieved while hauled out on land (Paterson et al. 2012). By increasing perfusion of warm blood to the skin surface, proliferation of skin cells and hair follicle development is optimised while at the same time increasing the supply of essential nutrients and oxygen for synthesis of skin and hair (Ling 1970). The thermal conductivity of water is approximately 25 times greater than in air (Nadel 1984) meaning that to maintain a high skin temperature while at sea is energetically prohibitive (Boily 1995; Watts 1996). There is therefore a clear requirement for phocid seals to be on land while moulting. Phocids that undergo a catastrophic moult such as the southern elephant seal (*Mirounga leonina*) complete the entire moult cycle in as little as 25–30 days (Boyd et al. 1993) whereas a longer, more diffuse moult is observed in other species such as the grey seal (*Halichoerus grypus*) (Boily 1996) and the harbour seal (*Phoca vitulina*) (Ashwell-Erickson et al. 1986).

The moult represents an important seasonal change in the behaviour and physiology of juvenile and adult phocid seals (Pearson et al. 2022; Thometz et al. 2023). However, these changes are often difficult to quantify due to the impracticalities of using loggers and/or telemetry devices in moulting animals—devices that are glued to the hair are lost when animals start moulting. Similarly, when recording physiological parameters such as heart rate, externally mounted electrodes normally require to be held in place against shaved patches of skin by glueing to surrounding hair (Fedak et al. 1988; Thompson and Fedak 1993; Ponganis et al. 1997). However, such devices cannot be used during the moult when old hair is being shed, and new hair is not strong enough to support device attachment. Harnesses have been used to attach loggers to otariid species but only in laboratory settings and only over periods up to five days during controlled experiments measuring activity with accelerometers (Dalton et al. 2014). To date, harnesses have never been successfully used in studies of phocids. The challenges of device attachment during the moult has resulted in a knowledge gap for this important stage of the lifecycle. Alternative methods are therefore required to maximise the quality and quantity of physiological data collected in moulting animals.

Heart rate and body temperature have been measured using fully implantable devices in a range of birds and mammals (Butler et al. 2004; Green et al. 2009; White et al. 2013; Wascher 2021), both in captivity (Mills et al. 2000; Dawson 2017; Sakai et al. 2024) and in the field (Bevan et al. 1997; Laske et al. 2011, 2021). However, research involving pinnipeds has seldom used fully implantable devices. In harbour seals, subcutaneously implanted VHF transmitters have been successfully used to track animal movements over periods of up to four years covering multiple moult periods (Blundell et al. 2014). Intraperitoneally implanted devices have also been designed to record abdominal body temperature over the lifespan of Steller sea lions (*Eumetopias jubatis*) (Horning and Mellish 2014). The studies by Blundell et al. (2014) and Horning and Mellish (2014) demonstrate that implantable devices can be used long-term in pinnipeds while not affecting the survival of individual study animals. As implantable loggers have become smaller, measurement of heart rate and body temperature is now possible in phocids (Chaise et al. 2017; Le Nabec et al. 2024). Such studies demonstrate that fully implantable devices can be used to measure physiological and behavioural parameters in pinnipeds that include the moult.

The aim of this study was twofold: firstly to assess the feasibility of measuring heart rate and T_b_ in captive harbour seals using a fully implantable logger. A long-term deployment on the scale of several weeks in a captive facility allowed for assessment of post-surgery healing after implantation and retrieval of loggers. This was important when considering potential deployment of such devices in the wild. The second aim was to compare heart rate and body temperature when animals were hauled out on land and in the water during moult and post-moult periods, providing further data on the physiology of moult in pinnipeds during this important stage of the annual lifecycle.

## Materials and methods

### Animals

Two male harbour seals, one adult and one sub-adult, were captured at Ardersier, Scotland (57°35’N, 04°02’W) on 22/05/2015 and transferred to the captive facility at the Scottish Oceans Institute, University of St. Andrews (UoSA). Animals were housed in outdoor pools in ambient temperature seawater and fed a varied fish diet supplemented with multivitamins and ferrous gluconate (Aquavits, International Zoo Veterinary Group, Keighley, UK). Each individual was weighed (± 0.1 kg) upon capture, opportunistically throughout the experimental period, and prior to release from the captive facility on 16/11/2015. All animal experiments were conducted under UK Home Office Licence (60/4009 and 60/7806) issued after initial review by the UoSA Animal Welfare Ethical Review Body.

### Heart rate/temperature logger

**Fig. 1:**
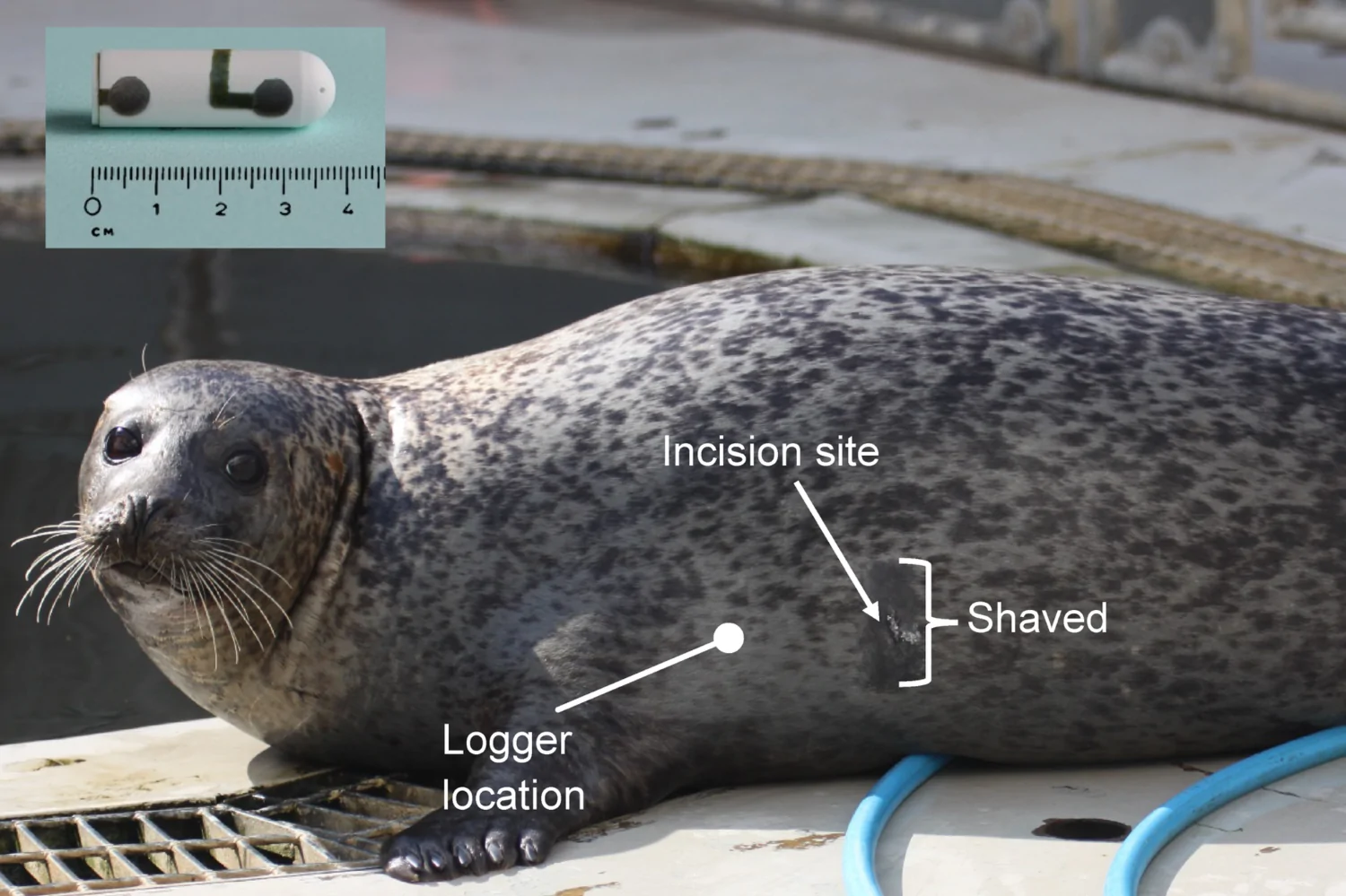
Location of DST milli-HRT logger (inset) relative to incision site. Image of adult harbour seal three days post-surgery on 21/08/2015.

An implantable logger (DST milli-HRT, Star Oddi Ltd., Iceland) was used for measuring heart rate (HR bpm) and sub-blubber body temperature (T_b_ ± 0.2 °C) at a predefined sampling interval of once every two minutes. Surgical implantation of loggers for the adult and sub-adult took place on the 18/08/2015 and 19/08/2015 respectively. The loggers were 13 mm diameter x 39.4 mm long cylinders weighing 11.8 g (Fig. 1). HR and sub-blubber body temperature were recorded between the hours of 17:00 and 00:00 when animals were most likely to voluntarily haul out on land. HR measurements were taken in burst recordings over the duration of approximately four to six heart beats once every two minutes. Specialist software (Mercury, Star-Oddi Ltd., Iceland) was then used to calculate mean HR based on interbeat intervals during each burst recording. The same software was used to assign a quality index (QI) from zero to three to each measurement (0 = Great, 1 = Good, 2 = Fair and 3 = Poor). QI was based on the level of signal to noise ratio (SNR) during measurements. Signal is the amplitude of the QRS waveform which is a description of the three wave types Q, R and S that correspond to the different stages of ventricular depolarisation during a heartbeat, while noise is the level of electrical activity between waveforms. A high SNR resulted in QI = 0 and a low SNR resulted in QI = 3.

### Surgical protocols

#### Anaesthesia

Both implantation and retrieval of implantable loggers were carried out under general anaesthesia. Seals were manually restrained before being mildly sedated with midazolam (0.05 mg/kg) via an intramuscular (IM) injection prior to general anaesthesia. Animals were left in a quiet area for 10 min, after which ketamine (1 mg/kg) was administered via intravenous (IV) injection to achieve general anaesthesia. The plain of general anaesthesia was maintained throughout surgeries via further IV injections of ketamine and midazolam as required. HR and blood oxygen levels were monitored throughout surgery using a pulse oximeter inserted in the rectum of animals (VM-2160 Pulse Oximeter, Viamed Ltd., UK, and a UYB rectal Pulse Oximetry Sensor, Thames Medical Ltd., UK).

### Implantation of loggers

All surgical procedures were undertaken in a purpose built, mobile surgical suite in a closed room adjacent to the seal holding tanks. The site of implantation of devices was located on the left flank approximately 15 cm posterior to the tip of the left fore-flipper (Fig. 1). This site was determined using a stethoscope to ensure that the apex beat projection area could be detected while also trying to avoid placement on the ventral surface where post-surgery abrasion may occur. The apex beat projection area is the point on the body surface where the maximum cardiac impulse can be heard using a stethoscope. A 10 x 5 cm area of skin approximately 10 cm posterior to the intended placement of devices was shaved and cleaned using iodinated povidone 7.5% surgical scrub (Vétédine^®^) and iodinated povidone 10% solution (Vétédine^®^). Three 1 ml boluses of a local anaesthetic (Lignol^®^ 2% w/v) were then injected along the implantation route at the blubber muscle interface with a further 1 ml bolus injected in the subcutaneous layer at the intended incision site. Animals were placed in a right lateral decubitus position (Lander et al. 2005) with sterile drapes used to cover the area surrounding the incision site. A 2 cm vertical incision was made. Non-traumatic Mayo scissors were then used to dilacerate tissue in the blubber layer until reaching the sub-blubber layer, here defined as the layer where the blubber layer interfaces with muscle tissue. Dilaceration continued along the sub-blubber layer interface anterior to the incision site creating a tunnel approximately 10 cm long. The DST-milli HRT was then inserted and pushed to the end of the dilacerated tunnel. A singular simple interrupted monofilament absorbable suture (PDS^®^ II D2T, 3 − 0, 3/8, 24 mm; Ethicon) was then applied in the deep adipose tissue to close the tunnel. Buried monofilament absorbable sutures (PDS^®^ II D3T, 2 − 0, 3/8, 24 mm; Ethicon) apposed the skin and subcutaneous incision edges before applying butyl cyanoacrylate tissue adhesive (Surgibond^®^) to the sutured incision site. The surgical wound was given a final clean with iodinated povidone 10% solution (Vétédine^®^) and then sprayed with Alumisol^®^ to protect the incision site and aid healing.

### Monitoring and retrieval

Post-surgery animals were monitored with daily visual inspections to assess progression of wound healing. Antibiotics (Baytril; 1.7 mg/kg) were administered orally for five days to prevent post-surgery infection. As part of visual inspections, a thermal camera (ThermaCAM™ P65, FLIR Systems Ltd., UK) was used to look for signs of infection as indicated by a persistent elevation in temperature around the wound site (Paterson et al. 2011). Loggers were retrieved using the same surgical protocol as for implantation with the exception that the incision site and dilaceration of tissue in the blubber layer was directly over the site where loggers were situated. The exact location of loggers was determined using ultrasound prior to choosing the incision site for retrieval. Surgical removal of devices for the adult and sub-adult animals was carried out on the 22/10/2015 and 23/10/2015 respectively, resulting in a deployment time of 65 days for both animals. Data was retrieved from the logger using the proprietary communication device and software connected by USB to computer.

### Measurement of haulout and in-water activity

During experimental recordings, animals were housed in a purpose-built, enclosed haulout respirometry chamber used for other concurrent studies (see Paterson et al. 2021 for details of experimental setup), allowing access to an underwater cage through an opening in the floor. Haulout and in-water activities were recorded using a closed-circuit video surveillance system with cameras (IR 37CSHR-IR 2 M Submersible, RF Concepts Ltd., Dundonald, UK) installed within the haulout chamber. Video was recorded using a digital video recorder (Samsung SRD-470, Hanwah Techwin America, New Jersey, USA) located inside the facility building meaning that seals were unaware of any human presence during experiments.

The term ’haulout event‘ refers to when approximately half of an animal’s body had exited the water and ended when approximately half of the animal’s body entered the water at the end of the haulout. Similarly, the term ’in-water event‘ refers to when approximately half of an animal’s body entered the water with animals remaining submerged and only periodically surfacing to breathe at the opening in the haulout chamber floor, ending when approximately half of the animal’s body had exited the water when hauling out again. Measurements of haulout events were recorded up to a maximum of 180 min after exiting the water and up to a maximum of 30 min after entering the water for in-water events.

### Environmental measurements

Air temperature (± 0.1 °C) was recorded inside the haulout chamber using a temperature logger (Tiny Tag Plus 2 TGP-4500, Gemini Data Loggers Ltd., West Sussex, UK). Water temperature (± 0.1 °C) of the experimental pool was also recorded using a thermistor and logger (Tiny Tag Plus 2 TGP-4020, Gemini Data Loggers Ltd., West Sussex, UK). In both devices, data were logged at a five-minute sampling interval.

### Moult categorisation

Animals were observed daily to determine the peak of the moult which was judged to be the week in which the amount of hair being shed during haulouts was at its maximum. Maximal hair loss was evident in the amount of hair found within the haulout chamber after experiments had been run. Determining the progress of shedding and renewal of hair in moulting harbour seals can be difficult due to the underlying processes not being visually apparent (Ashwell-Erickson et al. 1986). To eliminate subjectivity when deciding when the moult started and ended, moult duration was assumed to be a fixed period of 16 days either side of peak moulting in each animal, which is a maximum moult duration observed previously in harbour seals (Thompson and Rothery 1987). HR and temperature measurements recorded during these periods were categorised as moult measurements. All measurements after that time were categorised as post-moult measurements.

### Statistical analyses

#### Heart rate

HR (bpm) data were analysed in four separate models with a Linear Mixed Effect Model (LME) approach using the lme function in the nlme library in R (Pinheiro et al. 2017). HR values were natural log-transformed before modelling to reduce heteroscedasticity and skewness and to improve adherence to assumptions of normally distributed residuals and homogeneity of variance. Changes in HR with time during both haulout and in-water events, were modelled for the sub-adult and adult individually. Moult and post-moult periods were compared by including moult stage as a two-level factor while simultaneously fitting separate regression lines of HR at both levels. The full version of each model also included interaction terms between either minutes after haulout or minutes in water and whether animals were in a moult or post-moult state. When modelling HR over minutes hauled out, air temperature within the haulout chamber was included as a continuous explanatory variable. When modelling HR over elapsed time in water, water temperature was included as a continuous explanatory variable. In all models, repeated measures within each haulout or in water event were treated as a random effect to account for non-independence of measurements. Model selection was by stepwise backwards selection under maximum likelihood (ML) estimation. Competing nested models were compared using Akaike’s Information Criterion (AIC) and likelihood ratio tests (LRTs), with the most parsimonious candidate models being chosen based on improvement in model fit and whether AIC score reduction was significant. Final selected models were subsequently refitted using restricted maximum likelihood (REML) for parameter estimation.

#### Sub-blubber temperature

T_b_ data were analysed in four separate models with a Linear Mixed Effect Model (LME) approach using the lme function in the nlme library in R. Changes in T_b_ with time during both haulout and in-water events, were modelled for the sub-adult and adult individually. Moult and post-moult periods were compared by including moult stage as a two-level factor while simultaneously fitting separate regression lines of T_b_ at both levels. The full version of each model also included interaction terms between either minutes after haulout or minutes after in-water events and whether animals were in a moult or post-moult state. When modelling T_b_ over minutes after haulout, air temperature within the haulout chamber was included as a continuous explanatory variable. When modelling T_b_ over elapsed time in-water, water temperature was included as a continuous explanatory variable. In all models, repeated measures within each haulout were treated as a random effect to account for non-independence of measurements. Model selection was by stepwise backwards selection under ML estimation. Competing nested models were compared using AIC and LRTs, with the most parsimonious candidate models being chosen based on improvement in model fit and whether reduction in AIC score was significant. Final selected models were subsequently refitted using REML for parameter estimation.

All statistical analyses were carried out using the statistical package R (R Development Core Team 2025).

## Results

### Post-surgery monitoring

Both animals displayed similar patterns of recovery in terms of healing at the incision sites for implantation and retrieval. Over the first three to five days post-surgery, incision sites showed signs of granulation of tissue as well as slight swelling around sutures that was considered a normal part of the healing process. By five to seven days post-surgery, swelling around the sutures had largely receded and the incision sites had begun to close over. By 14 days post-surgery, incision sites were fully closed over and were largely indistinguishable from the surrounding skin and hair. In general, neither animal showed any sign of inflammation around incision sites during post-surgery healing. This indicated that the asepsis techniques during surgery as well as the antibiotic regime administered post-surgery were sufficient to allow a normal healing response. Data used for analyses in this study commenced on 25/08/2015, after both animals had fully recovered from surgery.

### Study animals and activity

Mean ± SE mass of the adult male used in this study was 92.9 ± 0.05 kg and 76.4 ± 0.21 kg during the moult and post-moult study periods respectively. The mass of the sub-adult male was 63.8 ± 0.08 kg and 61.1 ± 0.08 kg during the same periods (Table 1). Between 25/08/2015 and 16/10/2015, a total of 99 haulout events were recorded during 33 experiment days. Of those haulouts, 55 (adult = 29, sub-adult = 26) were during the moult and 44 (adult = 36, sub-adult = 8) during the post-moult. A total of 286 in-water events were recorded over 34 experiment days with 141 recorded during the moult (adult = 35, sub-adult = 106) and 145 (adult = 83, sub-adult = 62) during the post-moult.

**Table 1:** Mean ± SE mass (kg), and weighted mean heart rate (HR bpm) and sub-blubber temperature (T_b_ °C) during haulout and in-water events for the sub-adult and adult harbour seal during moult and post-moult periods

Animal	Period	Mass (kg)	Haulout		In water	
			Heart rate (bpm)	T_b_ (°C)	Heart rate (bpm)	T_b_ (°C)
Sub-adult	Moult	63.8 ± 0.08	87.4 ± 3.40	36.1 ± 0.13	74.4 ± 2.30	35.0 ± 0.08
	Post-moult	61.1 ± 0.08	90.2 ± 4.71	34.5 ± 0.57	93.8 ± 2.85	32.3 ± 0.07
Adult	Moult	92.9 ± 0.05	68.4 ± 2.80	35.8 ± 0.08	79.4 ± 3.72	35.6 ± 0.07
	Post-moult	76.4 ± 0.21	78.5 ± 2.79	33.3 ± 0.24	90.0 ± 1.91	32.1 ± 0.09

### Air and water temperatures

Mean air temperature was greater (t = 23.85, *p* < 0.001) during the moult (16.8 ± 0.05 °C) than during the post-moult (13.5 ± 0.05 °C) study period. Similarly, water temperature measured concurrently during the same periods was greater (t = 40.09, *p* < 0.001) averaging 14.5 ± 0.02 °C and 12.3 ± 0.03 °C during the moult and post-moult study periods respectively.

### Heart rate and sub-blubber temperature measurements

A total of 2127 and 2598 h and temperature measurements were recorded concurrently by the DST milli-HRT logger for the sub-adult and adult respectively during experiments in this study. All data were retained for analyses of T_b_ for both animals, however, based on recommendations by Star Oddi Ltd., HR measurements with the lowest QI index of 3 were discarded due to having a high SNR. Based on this criterion, 27% and 25% of HR data were discarded, resulting in 1555 and 1936 measurements being used for the sub-adult and the adult respectively in the final analyses. In each of the categories used during analyses, the final number of heart rate measurements used for the sub-adult were as follows; moult haulout (662; 19.4% discarded due to QI = 3), post-moult haulout (289; 32.1% discarded due to QI = 3), moult in-water (358; 24.8% discarded due to QI = 3) and post-moult in-water (246; 24.0% discarded due to QI = 3). For the adult, these were moult haulout (552; 19.5% discarded due to QI = 3), post-moult haulout (875; 25.0% discarded due to QI = 3), moult in-water (97; 32.1% discarded due to QI = 3) and post-moult in-water (412; 30.1% discarded due to QI = 3).

HR/temperature logger measurements recorded in each of the moult and post-moult study periods are summarised in Table 1 as weighted means, calculated at the event level to account for unequal numbers of measurements among haulout and in-water events. Calculated in this way, mean ± SE values represent averages across events in each category rather than individual logger measurements.

### Heart rate during haulout events

#### Sub-adult

During LME model selection for the sub-adult, the interaction term between minutes after haulout and moult stage (moult or post-moult) was not significant (LRT: χ²=1.32, *p* = 0.25) and so was removed. However, minutes after haulout was retained in the final model (χ²=23.67, *p* < 0.001), as was air temperature (χ²=4.49, *p* = 0.03).

**Fig. 2:**
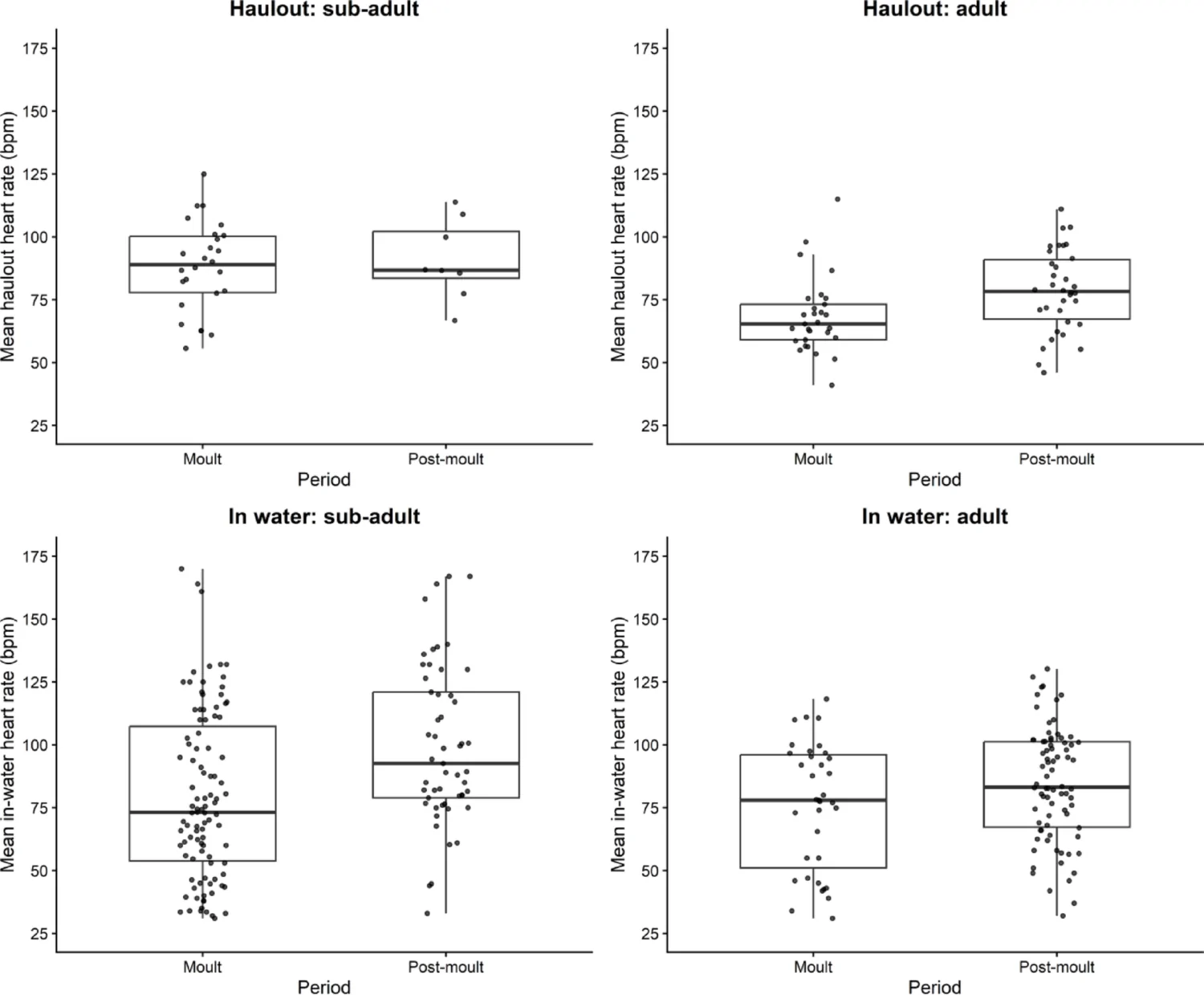
Event-level mean heart rate (bpm) for subadult moult haulout (*n* = 26), sub-adult post-moult haulout (*n* = 8), subadult moult in water (*n* = 106), sub-adult post-moult in water (*n* = 62), adult moult haulout (*n* = 29), adult post-moult haulout (*n* = 36), adult moult in water (*n* = 35) and adult post-moult in water (*n* = 83). Each point represents the mean heart rate calculated for an individual haulout or in-water event. Boxplots show the median, interquartile range and whiskers extending to 1.5 x the interquartile range

In the final LME, HR increased significantly with air temperature (β = 0.032 ± 0.015 SE, t_915_=2.10, *p* = 0.04) and minutes after haulout (β = 0.0012 ± 0.00024, t_915_=4.88, *p* < 0.001). HR during haulout events did not differ significantly between the moult and post-moult period after accounting for these covariates (β = 0.051 ± 0.081, t_32_=0.63, *p* = 0.54). Back-transformation of model coefficients indicated that HR increased by approximately 0.12% per minute following haulout and by approximately 3.2% for each 1 °C increase in air temperature. Figure 2 shows event-level mean HR values and their distribution for the sub-adult during haulout events in the moult and post-moult periods. Supplementary Figure S1 shows raw data for HR over minutes after haulout.

#### Adult

During LME model selection for the adult, the interaction term between minutes after haulout and moult stage (moult or post-moult) was not significant (LRT: χ²=0.31, *p* = 0.58) and so was removed. Subsequent model selection also showed that neither air temperature (χ²=2.96, *p* = 0.09) nor minutes after haulout (χ²=0.30, *p* = 0.59) were significant, which were also removed.

In the final LME, HR during haulout events differed significantly between the moult and post-moult periods, with HR being higher during the post-moult period compared with the moult period (β = 0.179 ± 0.043, t_61_=4.20, *p* < 0.001). Back-transformation of model coefficients indicated that HR during haulout events was approximately 20% higher post-moult compared with the moult.

Figure 2 shows event-level mean HR values and their distribution for the adult during haulout events in the moult and post-moult periods. Supplementary Figure S1 shows raw data for HR over minutes after haulout.

### Heart rate during in-water events

#### Sub-adult

During LME model selection for the sub-adult, the interaction term between minutes in water and moult stage (moult or post-moult) was not significant (LRT: χ²=1.06, *p* = 0.30) and so was removed. Subsequent model selection also showed that neither water temperature (χ²=2.45, *p* = 0.12) nor minutes in water (χ²=1.09, *p* = 0.30) were significant, which were also removed.

In the final LME, HR during in-water events differed significantly between the moult and post-moult periods, with HR being higher during the post-moult period compared with the moult period (β = 0.260 ± 0.059, t_151_=4.40, *p* < 0.001). Back-transformation of model coefficients indicated that HR during in-water events was approximately 30% higher post-moult compared with the moult.

Figure 2 shows boxplots of mean HR during in-water events for the sub-adult during the moult and post-moult periods. Supplementary Figure S2 shows raw data for HR over time in water indicating that variability of data in both the moult and post-moult periods was high which might explain why HR over minutes in water was not significant.

#### Adult

During LME model selection for the adult, the interaction term between minutes in water and moult stage (moult or post-moult) was not significant (LRT: χ²=0.001, *p* = 0.99) and so was removed. Subsequent model selection also showed that water temperature (χ²=2.08, *p* = 0.15) and minutes in water (χ²=3.27, *p* = 0.07) were non-significant, and removed.

In the final LME, HR during in-water events differed significantly between the moult and post-moult periods, with HR being higher during the post-moult period compared with the moult period (β = 0.121 ± 0.059, t_111_=2.04, *p* = 0.044). Back-transformation of model coefficients indicated that HR during in-water events was approximately 13% higher post-moult compared with the moult.

Figure 2 shows boxplots of mean HR during in-water events for the adult during the moult and post-moult periods. Supplementary Figure S2 shows raw data for HR over time in water indicating that variability of data in both the moult and post-moult periods was high which might explain why HR over minutes in water was not significant.

## Sub-blubber temperature

### T_b_ during haulout events

#### Sub-adult

During LME model selection for the sub-adult, the interaction term between minutes after haulout and moult stage (moult or post-moult) was retained in the final model (LRT: χ²=46.47, *p* < 0.001). Also retained in the final model was air temperature (χ²=62.51, *p* < 0.001).

**Fig. 3:**
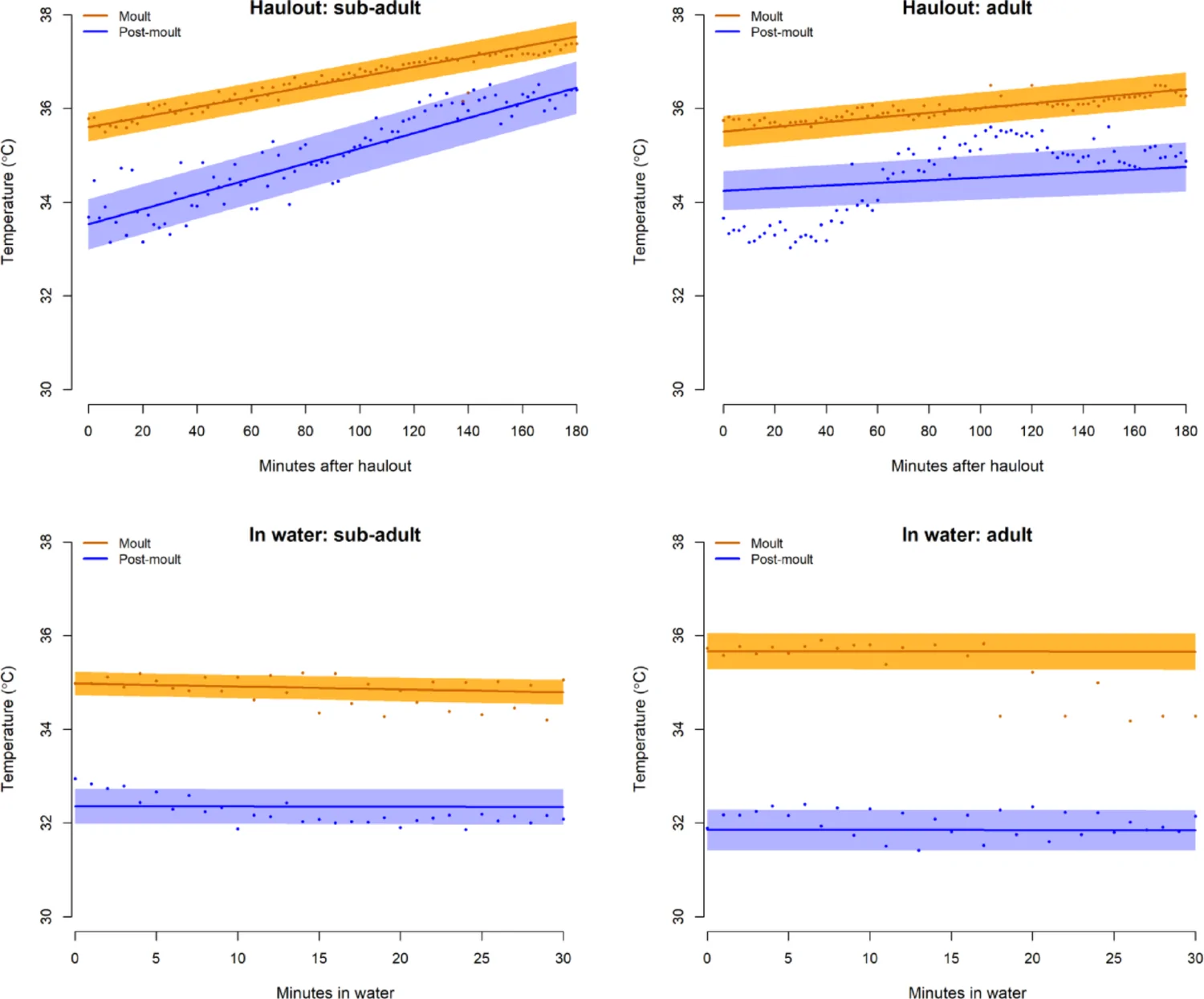
Linear mixed effect model (LME) predictions of sub-blubber temperature (T_b_ °C) for subadult and adult harbour seal after haulout and during in-water events (solid lines), for the moult (orange) and post-moult (blue) periods. Lines with 95% confidence intervals (filled areas) and mean temperatures given at each time point

In the final LME, T_b_ increased significantly with increasing minutes after haulout (β = 0.0107 ± 0.00045, t_914_=23.64, *p* < 0.001), while increasing air temperature was associated with significantly higher T_b_ (β = 0.113 ± 0.029, t_914_=3.94, *p* < 0.001). T_b_ during the post-moult period was significantly lower than during the moult period (β=−1.94 ± 0.31, t_32_=−6.21, *p* < 0.001). However, the significant interaction between minutes after haulout and moult stage indicated that T_b_ increased more rapidly during the post-moult period (β = 0.0055 ± 0.00080, t_914_=6.88, *p* < 0.001). Results for the sub-adult T_b_ LME model predictions for T_b_ during haulout are summarised in Fig. 3.

### Adult

During LME model selection for the adult, the interaction term between minutes after haulout and moult stage (moult or post-moult) was retained in the final model (LRT: χ²=11.77, *p* < 0.001). Also retained in the final model was air temperature (χ²=84.09, *p* < 0.001).

In the final LME, T_b_ increased significantly with increasing minutes after haulout (β = 0.0050 ± 0.00047, t_1359_=10.78, *p* < 0.001), while increasing air temperature was associated with significantly higher T_b_ (β = 0.169 ± 0.025, t_1359_=6.77, *p* < 0.001). T_b_ during the post-moult period was significantly lower than during the moult period (β=−1.99 ± 0.24, t_61_=−8.42, *p* < 0.001). However, the significant interaction between minutes after haulout and moult stage indicated T_b_ increased more slowly during the post-moult period (β=−0.0022 ± 0.00064, t_1359_=−3.47, *p* < 0.001). Results for the adult T_b_ LME model predictions for T_b_ during haulout are summarised in Fig. 3.

### T_b_ during in-water events

#### Sub-adult

During LME model selection for the sub-adult, the interaction between minutes in water and moult stage (moult or post-moult) was retained in the final model (LRT: χ²=5.91, *p* = 0.02). Also retained in the final model was water temperature (χ²=22.02, *p* < 0.001).

In the final LME, T_b_ during the post-moult period was significantly lower than during the moult period (β=−1.25 ± 0.25, t_203_=−5.05, *p* < 0.001). during the moult, T_b_ decreased significantly with increasing minutes in water (β=−0.0062 ± 0.00181, t_853_=−3.45, *p* < 0.001), with increasing water temperature associated with significantly higher T_b_ (β = 0.621 ± 0.125 SE, t_853_=4.98, *p* < 0.001). However, the significant interaction between minutes in-water and moult stage indicated that the rate of decline was reduced during the post-moult period (β = 0.0056 ± 0.00232, t_853_=2.43, *p* = 0.02). Results for the sub-adult T_b_ LME model predictions for T_b_ while in-water are summarised in Fig. 3.

#### Adult

During LME model selection for the adult, the interaction between minutes in water and moult stage (moult or post-moult) was not retained in the final model (LRT: χ²=0.63, *p* = 0.429). Having removed the interaction, minutes in-water did not significantly improve the model, and so was removed altogether (χ²=0.04, *p* = 0.846). Water temperature was retained (χ²=10.36, *p* = 0.001).

In the final LME, T_b_ increased significantly with increasing water temperature (β = 0.619 ± 0.183, t_601_=3.39, *p* < 0.001). T_b_ during the post-moult was significantly lower than during the moult period (β=−2.21 ± 0.41, t_128_=−5.42, *p* < 0.001). The model fit in this case was poor and did not fully meet the assumptions of a linear model. Results for the adult T_b_ LME model predictions for T_b_ while in-water are summarised in Fig. 3.

## Discussion

Measurement of HR of diving marine mammals has become feasible since the 1980s with the development of the digital electrocardiogram (ECG) logger through miniaturisation of electrodes, increased battery life and memory size, pressure-proofing and better attachment methods (Ponganis et al. 2021; Williams and Ponganis 2021). One of the challenges with external loggers is greater signal to noise ratio due to movement and effects of seawater on signal so implantation of loggers with leadless ECG derived HR has helped reduce this issue. Previous studies of pinnipeds have demonstrated the feasibility of long-term deployment of fully implantable data loggers (Horning and Mellish 2014; Manugian et al. 2015). However, in some cases devices have been prematurely removed (Green et al. 2009) or rejected (Blundell et al. 2014; Chaise et al. 2017) due to an acute inflammatory response to implantation. In this study, no inflammatory response was detected either after implantation or retrieval of loggers. Strict guidelines regarding asepsis (Lasa 2017) were adhered to during each surgical procedure which were performed in a purpose-built sterile surgical suite. By carrying out this study in a captive facility, animals could be monitored daily for signs of infection and/or inflammation. This also made it possible to administer a five-day post-operative antibiotic regime to reduce the risk of infection and a subsequent inflammatory response. Given a high standard of aseptic technique and animal husbandry practices, implantable loggers present a valuable technique to address research questions that may not be answerable using glue-on devices.

While animals were hauled out and in a resting state, HRs recorded in this study were comparable to predictions from allometric scaling of HR with body mass of aquatic mammals (He et al. 2023) (for adult = 84 ± 5.6 kg: allometric scaling = 76 ± 0.82 bpm, this study = 68–78 bpm; subadult = 62 ± 0.7 kg: allometric scaling = 80 ± 0.1 bpm, this study = 87–90 bpm. For comparison in a previous wild study of smaller hauled out harbour seals (male = 45 ± 5.6 kg: 101 ± 12.0 bpm; female = 44 ± 6.7 kg: 94 ± 2.8 bpm, Karpovich et al. 2015). For the adult during the moult, haulout HR was lower compared with the post-moult period. For the sub-adult, there was no difference in HR between the moult and post-moult periods when hauled out. Based on previous studies using HR as a proxy for metabolic rate (Boyd et al. 1999; Butler et al. 2004) a reduced HR during the moult indicates a lowered resting metabolic rate. Resting metabolic rate has indeed been shown to be lower during the moult in adult harbour seals (Ashwell-Erickson et al. 1986; Rosen and Renouf 1998) and northern elephant seals *Mirounga angustirostris* (Worthy et al. 1992). In contrast, an increased resting metabolic rate was measured in moulting grey seals (Boily 1996; Sparling et al. 2006), southern elephant seals (Boyd et al. 1993), and elevated in spotted (*Phoca largha*) and ringed seals (*Pusa hispida*) during moult (Thometz et al. 2023), suggesting possible inter-species variation in the energetic demands of moulting. The results of the present study indicate that as well as inter-species variation there may be evidence of differences in HR in moulting harbour seals dependent on age class, however, this could be due to variation between two individuals and larger sample size is needed.

During in-water events, HR was lower during the moult period compared with the post-moult period for both the adult and sub-adult. While it was the case that there were large fluctuations in HR while animals were in the water, this may reflect the fact that surface HR is likely to be two to three times higher than when animals are submerged (Fedak et al. 1988), meaning sharp increases or decreases will be recorded when seals surface or dive. Detailed analysis of diving behaviour was not assessed in this study, nevertheless, this variation did not appear to mask effects of moult on HR. Future studies should aim to combine physiological measurements with behavioural observations to determine how diving behaviour affects not only HR, but also T_b_ measurements.

T_b_ increased over time after haulout and moult temperature was higher at the point of hauling out and remained higher compared to the post-moult period up to three hours later for both animals. Moulting harbour seals increase perfusion of blood through anastomoses in the blubber layer while hauled out, increasing skin temperature to facilitate proliferation of hair follicle cells (Paterson et al. 2012). The results of the present study show an increase in T_b_ when hauled out, likely due to perfusion of blood through the blubber to the skin surface. Conversely, lower T_b_ measurements during the post-moult period were likely due to vasoconstriction of anastomoses in the blubber layer as animals conserved heat. Placement of loggers in the sub-blubber provided measurements of body temperature at the interface between muscle and insulating blubber. Low temperatures during the post-moult period suggests that the sub-blubber layer is affected by the gradient through the blubber layer (Hart and Irving 1959), possibly related to a reduction in blubber thickness. This was evident in both animals as the sub-adult decreased in mass from 63.8 to 61.1 kg, while the adult decreased from 92.9 to 76.4 kg between the moult and post-moult periods.

During the moult, the adult displayed relatively high T_b_ that remained high over the following 30 min during in-water events, suggesting that vasodilation of blood vessels at the sub-blubber layer may have continued while in the water. Harbour seals maintain skin temperature slightly above water temperature while submerged (Hart and Irving 1959) indicating that some blood flow to peripheral tissues is maintained even during diving. Increased control of vasodilation was evident for the adult during the post-moult period in that T_b_ during this time was already low upon entering the water and remained low thereafter, indicating that vasoconstriction in the blubber was more pronounced outside of the moult period. This pattern is consistent with the dive response whereby blood flow to peripheral tissues is reduced (Favilla et al. 2022). Low T_b_ at the point the adult entered the water during the post-moult may have been due to vasoconstriction occurring during haulouts as a means of conserving heat. It may also have been due to anticipation of entry into the water as part of the dive response, and an associated restriction of blood flow through anastomoses, demonstrated previously in harbour seals during training (Erdsack et al. 2012). These results suggest that at least in adult harbour seals, vasoconstriction in the blubber layer while in the water and during diving means that T_b_ is relatively independent of the surrounding environment. This is supported by the fact that there was no evidence of T_b_ changing over time after in-water events in either study period.

The sub-adult also displayed relatively high T_b_ while in the water during the moult compared with the post-moult study period. During the moult, T_b_ measurements decreased slightly over 30 min after in-water events, whereas little change was evident during the post-moult period. The T_b_ of the sub-adult also increased with water temperature. These results suggest that sub-blubber temperature in the sub-adult may have been influenced by water temperature during in-water events. Although the dive response would have reduced blood flow through the blubber layer, declining T_b_ during the moult, as well as increasing T_b_ with water temperature, suggest that vasoconstriction as part of the dive response was less effective at limiting heat transfer to the surrounding environment. Juvenile harbour seals with lower body mass have reduced survival over their first winter (Harding et al. 2005). A higher surface area to volume ratio as well as a reduced insulating blubber layer in smaller animals incurs a metabolic and/or hypothermic cost as a result of increased conductance across the blubber layer (Pearson et al. 2022). The decrease in T_b_ for the sub-adult while in the water and the effect of water temperature suggests that heat loss may have been relatively high in this individual when moulting.

## Conclusions

This study demonstrated that in a captive setting, the long-term deployment of a fully implantable HR/temperature logger can be achieved without complications. The surgical methods used in this study benefited from the relatively controlled conditions of the captive facility and careful post-surgical monitoring, but the same methods have been successfully used on southern elephant seals in the field (Chaise et al. 2017). The DST milli-HRT logger was able to detect fine scale changes in T_b_. This allowed insight into how peripheral blood flow to blubber and skin is controlled in adult and sub-adult harbour seals as they transition to and from the water both during the moult and after the moult is complete. Tb measurements suggested that peripheral perfusion increased during the moult, while measurements during in-water events were consistent with vasoconstriction associated with the dive response (Favilla et al. 2022; Le Nabec et al. 2024). In contrast, the variability in HR recordings was such that only large-scale differences between moulting and non-moulting animals could be observed. Overall, 25–27% of HR recordings were discarded due to poor quality. This discard rate is relatively high and is approximately 3–5 times higher than that seen in other studies on mammals using similar loggers (A Bjarnason 2016, pers. comm.). HR measurements that were discarded had high levels of electromyography (EMG) noise, possibly from muscle activity during movements. The use of implantable loggers is an important tool that can answer difficult physiological research questions. However, while the results of this study suggest that the DST milli-HRT logger was highly suitable for measuring body temperature in pinnipeds, improved methodology is required to improve the quality of HR data. Further optimisation of placement of loggers relative to the apex heartbeat and depth within the blubber layer, and new methods to filter out noise inherent in HR measurements will advance this method.

New developments in biologging since this study was undertaken have used several non-invasive techniques including acoustics (Day et al. 2017), photoplethysmography (Castaneda et al. 2018) and continuous-wave near-infrared spectroscopy to record heart rate in marine mammals (McKnight et al. 2021). Advances in wearable technology in human biomedicine (dos Santos et al. 2021) may now offer future opportunities for animal research without ethical and welfare issues from surgery. However, as shown in this study, logger implantation may still be required during life stages such as the moult and therefore finding new technological solutions for transmitting physiological measurements from within the body, to the skin surface which can then be remotely downloaded is an important aspiration for biologging (Williams et al. 2021). Future biologging on pinnipeds may therefore benefit from the successful surgical techniques used to implant loggers in this study.

## Supplementary Information

Below is the link to the electronic supplementary material.

Supplementary Material 1

## Data Availability

Data is available for download at: 10.5525/gla.researchdata.2053.

## Declarations

### GenAI Statement

GenAI (ChatGPT) was only used to assist with R coding and was not used in any other way.
