# Supplementary material for: Heart rate and sub-blubber temperature in harbour seals (Phoca vitulina) measured during the moult and post-moult using implantable loggers
Source: J Comp Physiol B. 2026 Jul 20;196(4):597–610. doi: 10.1007/s00360-026-01689-6 (PMC13506549; doi:10.1007/s00360-026-01689-6)
Supplement: Supplementary file 1 [file 360_2026_1689_MOESM1_ESM.docx]

**Supplementary Material**

**Heart rate and sub-blubber temperature in harbour seals (*Phoca vitulina*) measured during the moult and post-moult using implantable loggers**

Paterson, W. D., Chaise, L. L., Moss, S. E., Milne, R., Gilbert, C., Currie, J. I., Thompson, D., and McCafferty, D. J.*

**Supplementary Figure S1.** Mean±SE heart rate (bpm) over minutes after haulout for sub-adult moult (n = 662), sub-adult post-moult (n = 289), adult moult (n= 552) and adult post-moult (n = 875) categories. Note that data are presented at one-minute intervals as the logger, programmed to record every two minutes, gave a first recording either one or two minutes after haulout onset. Subsequent post-haulout recordings then occurred at two-minute intervals.

**Supplementary Figure S2.** Mean±SE heart rate (bpm) over minutes in water for subadult moult (n = 358), sub-adult post-moult (n = 246), adult moult (n = 97) and adult post-moult (n = 412) categories. Note that data are presented at one-minute intervals as the logger, programmed to record every two minutes, gave a first recording either one or two minutes after animals entered the water. Subsequent recordings then occurred at two-minute intervals.
